# Immunotherapy using Histobulin in atopic dermatitis

**DOI:** 10.1002/ccr3.3472

**Published:** 2020-11-11

**Authors:** Geunwoong Noh

**Affiliations:** ^1^ Department of Allergy and Clinical Immunology Cheju Halla General Hospital Jeju‐si Korea

**Keywords:** atopic dermatitis, histobulin (histaglobulin), immunotherapy, Non‐allergen‐specific allergy, upper respiratory infection

## Abstract

Histobulin was effective in atopic dermatitis. Allergic diseases may be classified as allergen‐specific and non–allergen‐specific. Histobulin appears to exert immediate preventive effects against upper respiratory infection.

## Graphical abstract

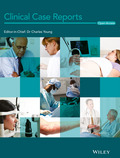

## 1 INTRODUCTION

Immunotherapy using Histobulin was attempted in four cases of atopic dermatitis. The patients showed considerable improvement, and one patient showed remission. The patients showed considerable improvement with a lower frequency of upper respiratory infections during immunotherapy.

Histobulin^TM^ (Green Cross PD) is a histamine‐fixed immunoglobulin preparation comprising 0.15 μg of histamine dihydrochloride and 12 mg of IgG.^1^ Histaglobulin^2^ and histaglobin^3^ are other generic names for Histobulin in other countries and reports. Histobulin was developed from histamine‐fixed serum, and it can inhibit antigen‐induced histamine release from human peripheral blood basophils and rat peritoneal mast cells.^4^, ^5^ The histamine from the Ig is assumed to induce antihistamine antibodies, which consequently provide resistance after allergen challenge.^2^ Histobulin is known to be effective in allergic rhinitis, chronic urticaria, and bronchial asthma.^2^, ^3^, ^6^ There are several reports finding that Histobulin is effective in atopic dermatitis.^7^, ^8^, ^9^, ^10^, ^11^, ^12^, ^13^, ^14^ In this report, immunotherapy using Histobulin was attempted in four cases of atopic dermatitis.

## 2 CASE REPORT

### 2.1 Case 1

A 19‐year‐old female patient visited the Department of Allergy and Clinical Immunology, Cheju Halla General Hospital, due to oozing and severe eczematous lesions mainly on the face with a history of atopic dermatitis. She had been treated with standard symptomatic therapy including antihistamines and steroids with persistent fluctuation of symptoms and signs. All patients in this report fulfilled the Haniffin and Rajka criteria.^15^ All four patients had no past medical history except atopic dermatitis from infancy. The clinical severity score was evaluated before and after treatment using SCORAD.^16^ The total score was 103 points. Basic allergic tests (blood tests and skin prick test) were conducted on all three patients before and after treatment. They received blood tests for a complete blood count with differential, serum eosinophil cationic protein, and serum total IgE and IgE levels for specific allergens using a multiple allergosorbent test (MAST, Green Cross PD). In the MAST test, the specific IgEs for 41 allergens were evaluated, including *Dermatophagoides pteronyssinus* (Dp), *D farina* (Df), cat, dog, egg white, milk, soybean, crab, shrimp, peach, mackerel, rye pollen, house dust mites, cockroach, *Clasporium herbarum*, *Aspergillus fumigatus*, *Alternaria alternata*, birch‐alder mix, white oak, short ragweed, mugwort, Japanese hop, hazelnut, sweet vernal grass, Bermuda grass, orchard grass, timothy grass, reed, *Penicillium notatum*, sycamore, sallow willow, poplar mix, ash mix, pine, Japanese cedar, acacia, oxeye daisy, dandelion, Russian thistle, goldenrod, and pigweed. The test results show the level of specific IgE for each allergen, and a normal negative range is 0.000‐0.349 IU/mL.

A skin prick test was also performed for 53 allergens. The allergens tested by the skin prick test were *A alternaria*, *A fumigatus*, *A nigre*, *Candida albicans*, *Cladosporium*, *P chrysogenum*, German cockroach, Dp, Df, dog, cat, grey elder/silver birch, grass mix, mugwort, short ragweed, black willow pollen, orchard grass, Bermuda grass, timothy, English plantain, English rye grass, Holm oak, Japanese cedar, cotton flock, milk mix, egg mix, chicken, beef, pork, cod, oyster, salmon, prawn, mackerel, tuna, almond, peanut, bean, carrot, cabbage, walnut, maize, peach, tomato, black pepper, spinach, wheat flour, rabbit, kapok, hop, F acacia, pine, and poplar. Skin prick tests are performed on the upper back between the scapular spine and L1 spine. The area to be tested was cleaned with alcohol and coded with a skin marker pen corresponding to the number of allergens being tested. The marks were 2 cm apart. A drop of allergen solution is placed beside each mark. A small prick through the drop is made to the skin using a Morrow Brown Needle^®^ (Morrow Brown^®^ Allergy Diagnostics) by holding the needle perpendicular to the test site and punching firmly through testing extract and into epidermis. The drop was removed immediately after the skin has been pricked, and the used needle was discarded immediately. Histamine hydrochloride 1 mg/mL was used as a positive control, and physiologic saline was used as a negative control. The results were measured as the wheal size. Reactions were read after 15 minutes and described as negative (0, no reaction), 1+ (reaction greater than control reaction but smaller than half the size of histamine), 2+ (equal to or more than half the size of histamine), 3+ (equal to or more than the size of histamine), and 4+ (equal to or more than twice the size of histamine). The minimum size of a positive reaction is 3 mm.

Patients received 2 mL of Histobulin (12 mg human immunoglobulin/0.15 μg histamine complex) by subcutaneous injection in the deltoid areas of upper arm every week for 24 or 36 times. All patients stopped to take antihistamine at least a week before Histobulin therapy.

**FIGURE 1:**
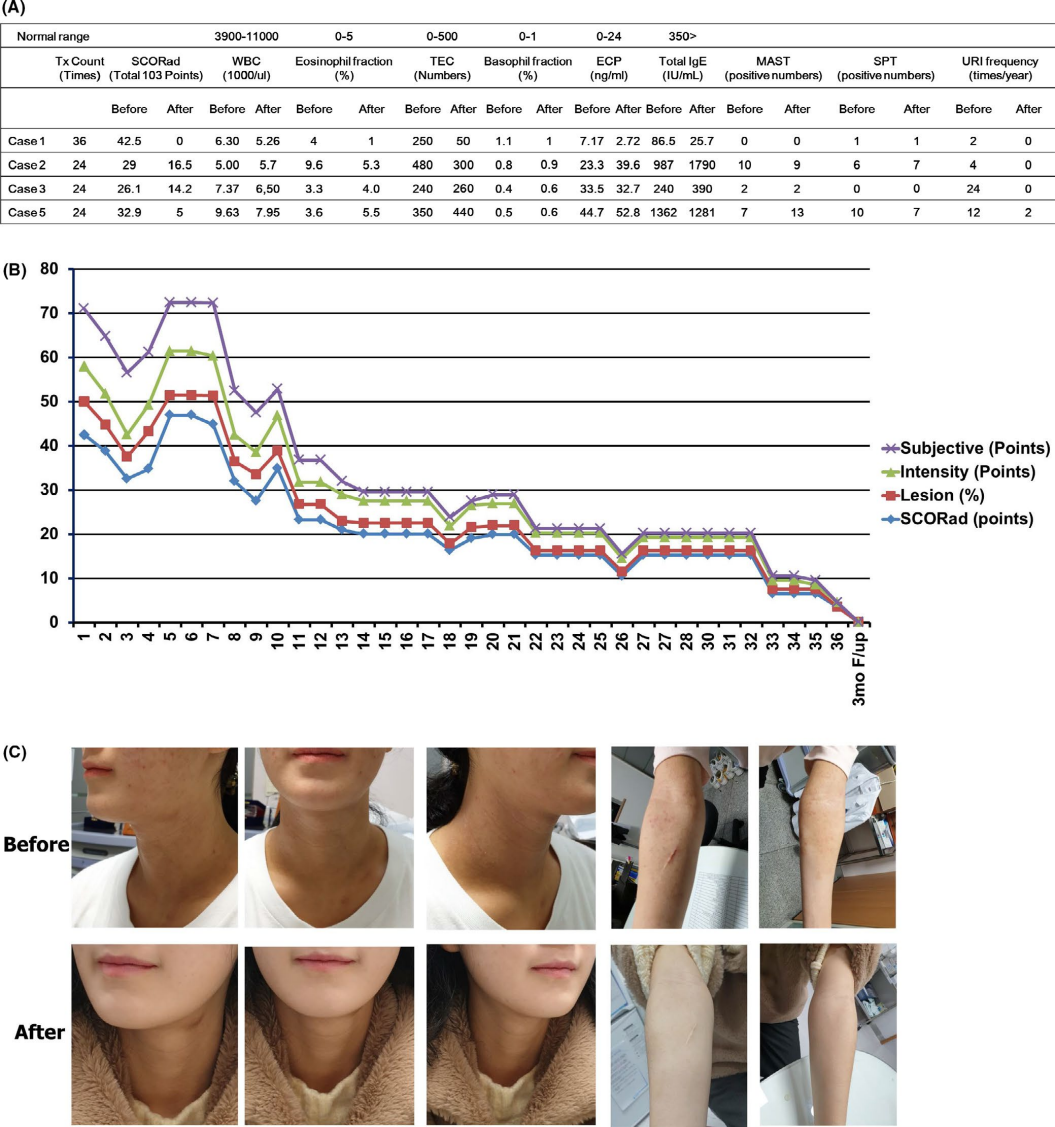
(A) The clinical severities, laboratory test results, skin test results, and frequency of URI before and after Histobulin therapy, (B) the clinical progress of case 1 during Histobulin therapy in atopic dermatitis. Case 1 improved initially. One aggravation episode followed the initial improvement; thereafter, the patient gradually improved, and the atopic dermatitis finally remitted; (C) before Histobulin therapy, the patient showed oozing, papular eruption, eczema, excoriation, and hyperpigmentation on the face, neck, and both upper extremities and the trunk, back, and lower extremities. After Histobulin therapy, all skin lesions subsided and were restored to rather fine skin

Her clinical severity was improved from 42.5 to 0 (points), and her atopic dermatitis was remitted clinically after 36 Histobulin injections. The clinical severity scores, laboratory test results, and skin test results before and after treatment are shown in Figure 1A. Her atopic dermatitis was remitted after 36 Histobulin injections (Figure 1B,C). In the MAST, specific IgE for allergens was not detected, and in the skin prick test, only Japanese cedar was positive as 2 + before and after treatment. The URI frequency was decreased, and she did not suffer from URI for 1 year including the treatment period.

### 2.2 Case 2

A 15‐year‐old male patient visited the clinic due to erythematous lesions on the face, neck, and elbow. He suffered from atopic dermatitis from infancy which had been more aggravated during the last year. He received standard treatment with persistent fluctuation. His clinical severity was improved from 29 to 16.5 (points) after 24 Histobulin injections. The laboratory and skin test results before and after treatment are shown in Figure 1A. The severity score of atopic dermatitis was improved gradually with 24 Histobulin injections. The results of the MAST showed 10 positive allergens (Dp 100<, Df 100<, milk 1.0, *Cladosporium* 0.69, ragweed short 6.95, Bermuda grass 0.79, orchard grass 2.49, timothy grass 0.36, *P notatum* 0.56, and Japanese cedar 14.19) before treatment, and 9 allergens were positive (Dp 100<, Df 100<, *C herbarum* 0.65, ragweed short 1.37, Bermuda grass 1.30, orchard grass 2.85, reed 0.64, *P notatum* 0.57, and Japanese cedar 5.14) after treatment. In the skin test, six allergens (Dp 4+, Df 4+, grass mix 2+, orchard 3+, timothy 2+, and English rye grass 3+) showed positive reactions before treatment, and 7 allergens (Dp 4+, Df 4+, grass mix 3+, orchard 3+, timothy 2+, English rye grass 4+, and Japanese cedar 3+) were positive after treatment. Before treatment, the patient suffered from URI 4 times per year, but he did not experience URI during or after treatment for a year.

### 2.3 Case 3

A 15‐year‐old male patient visited the clinic due to vesiculopapular eruptions on the face and neck. He suffered from atopic dermatitis also from infancy, but it was abruptly aggravated during the past week. He received standard treatment thereafter showing persistent fluctuation. He received Histobulin therapy through 24 injections, and the clinical severity scores improved gradually from 26.1 to 14.2 after 24 Histobulin injections (Figure 1A). The MAST results showed that 2 allergens were positive (*A alternata* 100 <and Dp 2.40) before treatment and 2 allergens were positive (*A alternata* 100 <and Dp 2.40) after treatment. In the skin test, no allergens showed a positive reaction before or after treatment. The patient frequently suffered from URI 24 times per year (two times per every month), but he did not experience URI during or after treatment for a year.

### 2.4 Case 4

A 20‐year‐old female patient visited the clinic due to eczematous lesions on the posterior neck, flexure area of the elbow, and popliteal area with known atopic dermatitis. She also suffered from atopic dermatitis from infancy. She received a standard treatment also without effectiveness, showing persistent fluctuation. Her clinical severity scores were improved from 32.9 to 5 after 24 Histobulin injections. The laboratory test results and skin test results before and after treatment are shown in Figure 1A. The MAST results showed that 7 allergens were positive (Dp 100<, Df 100<, shrimp 0.61, *Cladosporum hertiarum* 1.34, *A fumigatus* 2.65, *A alternata* 1.88, and mugwort 4.41) before treatment and 12 allergens were positive (Dp 100<, Df 100<, crab 0.41, shrimp 0., *C herbarum* 8.89, *A fumigatus* 5.10, Alternaria alternata 14.19, mugwort 1.13, *P notatum* 44.23, oxeye daisy 0.74, dandelion 0.57, and goldenrod 0.62) after treatment. In the skin test, 10 allergens (Dp 4+, Df 4+, grey Alder with silver birch 2+, mugwort 4+, short ragweed 2+, black willow pollen 2+, Japanese cedar 2+, milk mix 2+, almond 2+, and peanut 2+) showed a positive reaction before treatment and 7 allergens (*C albicans* 2+, Dp 4+, Df 4+, mugwort 4+, black willow pollen 3+, Japanese cedar 1+, and peanut 2+) after treatment. The patient frequently suffered from URI 12 times per year, but he did so only twice during and after treatment for a year.

## 3 DISCUSSION

Histobulin was very effective in atopic dermatitis in these cases. Actually, Histobulin therapy in allergic dermatoses has been attempted since 1968.^7^ Although there were several reports in that early period, the studies concerning the therapeutic effects of Histobulin in atopic dermatitis are very rare, and they were discontinued several decades ago.^8^, ^9^, ^10^, ^11^, ^12^, ^13^, ^14^

Histobulin inhibits NF‐kappa B nuclear translocation and downregulates proinflammatory cytokine.^17^ Histobulin inhibits allergen‐induced peritoneal accumulation of eosinophils in mice.^18^ In cases 1 and 2 in this report, the blood eosinophil fraction and total eosinophil counts were decreased. Additionally, Histobulin inhibits antigen‐induced histamine release from human peripheral blood basophils^4^, ^5^ and is possibly related to the improvement of atopic dermatitis.

Intravenous immunoglobulin (IVIG) provides a large quantity of immunoglobulin, and IVIG was also effective in treating atopic dermatitis.^19^ The immunoglobulins in Histobulin possibly played a role in the therapeutic effects of Histobulin in atopic dermatitis.

From a therapeutic perspective, Histobulin therapy is a non–allergen‐specific immunotherapy. Some cases showed no allergen sensitization. Thus, allergies might be classified as allergen‐specific allergies and non–allergen‐specific allergies. Histobulin therapy and IVIG therapy might be a type of non‐allergen‐specific immunotherapy for non–allergen‐specific allergy. IFN‐γ showed polydesensitization effects in allergen‐specific immunotherapy in atopic dermatitis.^20^ IFN‐γ also seems to have a non–allergen‐specific effect from the polydesensitization effect.

In terms of disease, Histobulin is also effective in chronic urticaria with no abnormal laboratory findings and no sensitization to allergens.^2^, ^3^ Based on this concept, chronic urticaria may be classified as a non–allergen‐specific allergy. One strategy for a therapeutic approach to allergic diseases is to classify allergic diseases as allergen‐specific and non–allergen‐specific.

Based on the action mechanisms of Histobulin, histamine is mediated in the pathogenesis of atopic dermatitis according to the results. It should be clarified whether IgE‐mediated allergy and non–IgE‐mediated pathogenetic mechanisms operate sequentially or independently. However, from the effects of Histobulin in this report, non–IgE‐mediated allergic reaction seems to be blocked by the blocking of IgE‐mediated allergic reaction by Histobulin.

Remarkably, all patients showed a lower frequency of upper respiratory infection during Histobulin therapy. Three patients showed no episode of upper respiratory infection during treatment. The important point is that they did not suffer from upper respiratory infection during therapy. One patient, case 3, suffered from upper respiratory infection twice every month, but he did not catch any upper respiratory infections just after beginning Histobulin therapy. Namely, the preventive effect for upper respiratory conditions was immediate just after beginning treatment. Histobulin has been used in the treatment of recurrent inflammatory states of the respiratory tract in atopic children.^21^ Moreover, Histobulin therapy is effective in the prevention of upper respiratory infections in children.^22^ The results of this report also support the evidence that Histobulin may be useful in preventing upper respiratory infection. One possible mechanism is the improvement of allergic conditions. Airway allergies increase the risk of viral infection.^23^ The improvement of allergic conditions possibly reduced the risk of respiratory viral infection. Another suspected mechanism is the possible preventive or therapeutic effects of immunoglobulin in histamine on viral infection. Histobulin is effective in viral hepatitis.^24^, ^25^ However, the exact mechanisms need to be investigated. It has even been applied in epidemic keratoconjunctivitis.^26^ Hence, Histobulin can be possibly applied as a preventive therapeutic for upper respiratory infection, at least in allergic patients.

## 4 CONCLUSIONS

Histobulin therapy is effective in atopic dermatitis as a non–allergen‐specific immunotherapy. Allergic diseases need to be classified as allergen‐specific and non–allergen‐specific, and a relevant therapeutic strategy is also necessary. Histobulin seems to exert immediate preventive effects for upper respiratory infection. Thus, Histobulin is suggested as a preventive pharmaceutical for upper respiratory infection nonvirus specifically.

## CONFLICT OF INTEREST

The authors declare that there is no conflict of interest regarding the publication of this manuscript.

## AUTHOR CONTRIBUTIONS

GN: is the only author of this manuscript.

## Data Availability

None.
